# Spectrophotometric Evaluation of Enamel Color Variation Using Infiltration Resin Treatment of White Spot Lesions at One Year Follow-Up

**DOI:** 10.3390/dj8020035

**Published:** 2020-04-10

**Authors:** Roberto Lo Giudice, Frank Lipari, Francesco Puleio, Angela Alibrandi, Fabrizio Lo Giudice, Cristina Tamà, Evgenia Sazonova, Giuseppe Lo Giudice

**Affiliations:** 1 Department of Clinical and Experimental Medicine, Messina University, 98100 Messina, Italy; 2 Department of Biomedical and Dental Sciences and Morphofunctional Imaging, Messina University, 98100 Messina, Italy; frank.lipari@hotmail.it (F.L.); francesco.puleio@live.it (F.P.); fabrizio.logiudice@hotmail.it (F.L.G.); cristinatama6@gmail.com (C.T.); genny.sazonova@gmail.com (E.S.); logiudiceg@unime.it (G.L.G.); 3 Department of Economics, Section of Statistical and Mathematical Sciences, Messina University, 98100 Messina, Italy; aalibrandi@unime.it

**Keywords:** white spot lesion, resin infiltration, enamel demineralization, aesthetics, camouflage effect

## Abstract

The aim of this study is to evaluate the color changes and the stability at a 1-year follow-up of white spot lesions (WSLs) treated with an infiltrating technique by using etching and TEGDMA resin. The color of 22 white spot lesions and the sound adjacent enamel (SAE) were assessed with a spectrophotometer at T0 (baseline), T1 (after treatment), and T2 (1 year after). The color change ΔE (WSLs-SAE) at T0 vs. T1 were compared to evaluate the camouflage effect efficiency, and at T1 vs. T2 to assess the stability of outcomes. To evaluate the effect on the treatment outcome of gender, the presence or not of previous orthodontic treatment, WSLs onset more/less than 10 years, the age of the patient, and the ΔE WSL (T0 vs. T1) was analyzed. The difference between ΔE (WSLs-SAE) at T0 and T1 resulted in statistical significance (*p* < 0.01). No statistical difference was found between ΔE (WSLs-SAE) at T1 vs. T2. The variables considered showed no statistical differences in treatment outcomes. The results of our investigation show that the technique used is immediately effective and the camouflage effect keeps up and steady one year after treatment. Such results do not appear to be influenced by analyzed clinical variables.

## 1. Introduction

Enamel translucency is a characteristic related to the enamel refractive index (RI = 1.62) and inter-crystalline space composition [1]. Demineralization alters the physiological enamel reflectivity, and the difference in RI between the healthy enamel and the demineralized area generates a milky white opaque appearance, clearly distinguished from the sound adjacent enamel (SAE) [2,3]. 

The enamel white lesion could be distinguished in fluorosis, opacities, or white spot lesions (WSLs) [4] that, in particular, have been described as hypomineralization, usually limited to anterior teeth [5], and could be caused from demineralization of enamel subsurface that might evolve into a carious lesion. However, the presence of a cavity modifies the diagnosis and transforms the treatment into a conventional restorative process.

A white spot lesion can be considered as part of the caries disease process, due to plaque accumulation, and influenced by other factors, such as diet, fluoride exposure, saliva properties, as well as genetic factors [5]. Some authors show how remineralization is possible in the initial phase of the lesion [6,7]; however, in patients with inadequate oral hygiene, the lesions, contrariwise, can progress and became a proper cavity, making subsequently restorative treatments necessary [8,9].

These lesions are found in about 46% of patients with fixed orthodontic treatments due to the presence of plaque around brackets and bands and to the greater difficulty in maintaining correct oral hygiene especially in the case of complex surgical-orthodontic treatments and/or an excessive extension of the enamel surface etching during the orthodontic bracket positioning [10,11].

The prevention and the evolution of WSLs mean they can be performed with non-invasive treatments, based on the use of remineralizing agents such as toothpaste containing 5% fluorine or remineralizing agents containing casein phosphopeptide amorphous calcium phosphate (CPP-ACP) [12,13,14,15,16,17]. However, this approach can only be applied in the early stages of WSL development; furthermore, remineralization does not occur in the whole area of the white spot, but only in the farthest and deepest portion of the surface, leaving it clinically visible to the naked eye and thus not solving the aesthetic problem [6,18,19], so more invasive approaches, such as direct restorations or preparations for indirect restorations that could result in a sacrifice of healthy dental tissue, could be needed.

For these reasons, a minimally invasive treatment, which, after etching with a 15% hydrofluoric (HCL) acid, increases white spot area porosity, consists of the infiltration of a highly viscous and penetrating resin in the thickness of WSLs that prevents lesion progression and inhibits caries progression [20]. Moreover, having a refractive index like the enamel one, it masks the opaque white appearance typical of WSLs [1,21,22].

The aim of this study is to evaluate the color (E) and gloss changes of WSLs treated with an infiltrating technique and the stability of the treatment over time. The color difference is expressed as ΔE values; the null hypothesis consists of no ΔE change when comparing WSL vs. SAE immediately after the treatment and a ΔE variation after one year.

## 2. Materials and Methods 

The research was conducted between 2017 and 2019 and included 22 patients (10 males, 12 females, ages between 12 and 29 years; mean 18.6 ± 4.7). From each patient, one lesion was selected and treated by the same calibrated operator. The inclusion criteria were the presence of one non-cavitated, unrestored WSL. The exclusion criteria were enamel alteration (fluorosis, opacity, hypocalcification, hypoplasia), the presence of enamel cavitation, reduced salivary flow, and tooth wear.

The sample size was determined with a type-I error = 0.05 and a power = 0.85 using parameter ΔE WSL vs. SAE after treatment. For each patient, information regarding sex, age, multibrackets orthodontic treatment (y/n), and time of lesion onset (more or less of 10 years) was collected.

The treatment of the white spots was carried out by applying an infiltrating acrylate resin (TEDMA 70%–95% and Camphoro Quinone < 2.5%) according to the operational steps suggested by the manufactory (DMG Chemisch-Pharmazeutische Fabrik GmbH Germany) summarized in Table 1:

The enamel color changes induced by WSLs treatment were analyzed by spectrophotometric examination (Spectroshade Micro Device; Medical High Technologies, Verona, Italy). This device defines in a three-coordinate diagram the color space using CIE-L*a*b*. L represents the brightness value in a range from 0 (black) to 100 (white) and a and b represent two ranges of colors ranging from green to red and from blue to yellow with values from −120 to +120.

The color change (ΔE) was calculated using the following formula:

ΔE (Par 1−Par 2) = [(LPar1−LPar2)^2^ + (αPar1−αPar2)^2^ + (βPar−βPar)^2^]^1/2^

The color was measured at 3 random points of the lesion, and the average of the values obtained was calculated [23]. For the SAE the color was measured in 3 points: 1 upper, 1 left, and 1 right to the lesion, at 2 mm from WSLs margin. For each patient, the observation times were (T0) before the application of the infiltrating technique, (T1) after the infiltrating technique, and (T2) after 12 months. At every observation time, an estimation of color difference (ΔE) between WSLs and SAE, which was considered as a control reference, was carried out. The ΔE identified at T0 and T1 were compared using the Wilcoxon test to evaluate the camouflage effect efficiency immediately after the resin infiltration treatment. The stability of outcomes one year after treatment was analyzed, observing the variation of color difference between WSLs and SAE in T1 and T2 using the Wilcoxon test. For all variables analyzed, the comparison of WSLs color before and after treatment (T0 vs. T1) was performed using the Mann–Whitney and Spearman tests, which allowed the analysis of the effect of the considered variables on the treatment outcome with *p*-value < 0.05 considered as significant. For statistical analysis, the SPSS software for Windows ver.22 (IBM, Milan, Italy) was used.

All subjects gave their informed consent for inclusion before they participated in the study. The study was conducted in accordance with the Declaration of Helsinki, and the protocol was approved by the Ethics Committee of 19-2018 of 23 April 2018.

## 3. Results

Briefly, 40.9% of patients had fixed orthodontic treatments, 59.1% of patients have never undergone any orthodontic treatment. The group of 40.9% of patients was referred to the WSLs onset for less than 10 years.

**Figure 1:**
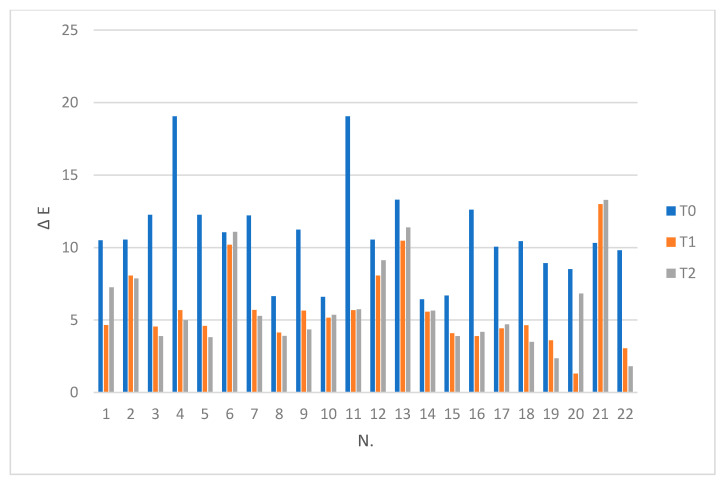
ΔE (WSL vs. SAE) at T0, T1, and T2 for each patient.

All patients (100%) were available at the 12-month follow-up. ΔE values recorded for WSLs vs. SAE at three observation times are described in Table 2 and Figure 1.

Comparing the color difference ΔE (WSLs-SAE) at T0 and T1, the Wilcoxon test pointed out the statistically significant difference (*p* < 0.01), while for ΔE (WSLs-SAE) at T1 and T2, the color variation was not statistically significant (*p* = 0.935). The color change comparison of WSLs between T0 and T1 is described in Table 2 and Figure 1. According to the Mann–Whitney test, there were not any statistically significant differences of WSLs color variation ΔE (T0 vs. T1) as regards sex (*p* = 0.468), previous fixed orthodontic treatment or no orthodontic treatment (*p* = 0.332), WSLs onset more/less than 10 years (*p* = 0.462). Two of the case analyzed (Samples 6, 21) showed a remarkable ΔE difference at T0, T1, and T2 when compared to the other samples. Both WSLs were not linked to orthodontic treatment. These results could be linked to an altered enamel morphology that could influence the tooth porosity and, so, the tooth color. 

In accordance with the Spearman test, there was no significant correlation between immediate color change efficacy ΔE (T0–T1) after treatment and patient age (*p* = 0.849).

## 4. Discussion

Many studies have demonstrated the effectiveness of the infiltrating technique in WSL treatment [1,21,22,24,25,26,27]. The technique used provides, in order to increase porosity, preliminary white spot area etching with HCl 15% for 2’ and subsequent resin infiltration. Some authors have reported that the number of etching applications can be correlated to WSL characteristics as wide, deep, smooth, and shiny lesions need more etching steps and might remain visible after resin infiltration [28,29]. The technique turns out to be minimally invasive, considering that the etching demineralisation of enamel is no more than 30 µm [30].

The resin used for infiltration is mainly composed by TEGDMA, a dimetacrylate characterized by high infiltration coefficient and refractory index like healthy enamel, (1.52 vs. 1.65); both characteristics allow the concealment of the WSLs [1,21,25]. 

It was observed that the treatment has some limitations in long term result stability if the resin used possesses a higher capacity of water absorption than other resins such as BisGMA and UDMA [31,32]. Such property has been correlated to pigmentation tendency due to the carrier effect of water for various pigments [33,34]. 

To prevent color alteration overtime, Borges et al. suggest polishing the treated surface before treatment; a more conservative alternative is to use the walking bleach technique with carbamide peroxide on treated lesions [23,35,36]. 

Considering that the dental color evaluations carried out using colorimetric scales are less precise, being dependent on the operator and subject to a variability linked to different observations during the time, in our study, the color evaluation was carried out using a spectrophotometer.

Many researchers have assess the immediate effect and stability over time of this treatment, comparing the color mean values in different observational groups [27,28,34]. Our methodological approach foresees the color difference assessment between WSL or treated lesions and SAE in every patient at a different observation time [37]. The control reference used was the sound enamel color, given its steady value, reliable measurement, and ease of use. Immediate treatment efficacy and stability were assessed statistically, analyzing the individual ΔE modifications using the Wilcoxon test. 

From the analysis of our data, it appears evident how our results agree with the data available from the literature, even if the methodology used differs. Our methodology, in our opinion, allows a more accurate statistical evaluation since it compares the ΔE variation in the same tooth, avoiding the comparison among groups with different color distributions.

The research data of the immediate effectiveness of the treatment demonstrate a highly significant variation of ΔE with decreased color differences between treated enamel and SAE. No significant differences found one year after treatment confirm that the technique used assures result stability for the period considered. The null hypothesis is rejected.

The statistical analyses of our categorical data highlight that variables considered by other studies as possible causes of less immediate treatment effectiveness (sex, multibrackets orthodontic treatment, and lesion onset more than 10 years) do not determine any statistically significant variation of results. Moreover, the Spearman test shows that patients’ age does not influence the result of the treatment. 

Our statistical data appears to be discordant with the results by Klaus W. Neuhaus, who advises infiltrating the lesions as soon as possible after the removal of fixed orthodontic treatment, avoiding that the lesion becomes smooth and shiny, and thus, more difficult for resin infiltration [29]. 

A low efficiency of this technique was detected in two cases that never underwent any orthodontic treatment, and, beside the absence of statistical relevance, this observation could be linked to a lower resin infiltration capability determined by low porosity in the after-etching area.

One of the major points of our research is that besides evaluating the treatment outcome for orthodontic patients only, we evaluated this kind of lesion in patients that never underwent any orthodontic treatments, also comparing all the clinical parameters and clinical outcomes and the long-term stability between the two groups.

Moreover, the long-term result of a therapy is an important factor in the decisional tree that leads to the performance of a therapy over another. Considering that this kind of treatment is aimed at correcting color and, therefore, is an aesthetic problem, the long-term stability of opacity/color is a very important factor.

A 6-month follow-up, as in many pieces of research, appears to be too short, considering that it is reported how TEGDMA possesses a higher capacity of water absorption than other resins such as BisGMS and UDMA, so a minimum of 12 months follow-up is suggested.

Moreover, the research methodology that is different from the cited paper appears, in our opinion, more precise, allowing a more accurate statistical evaluation since it compares the variation of ΔE in the same tooth, therefore, the dichromatism cloaking clinical effect. In our study, we avoided the use of color mean comparison, considering its low statistical/clinical significance determined by the different color distribution in the same tooth sample.

The limitation of the present study can be linked to the relatively small sample analyzed. Further study in this field could evaluate the evolution of these lesions to a longer follow-up.

## 5. Conclusions

The results of our investigation show that the ΔE (WSLs/SAE) at T0 and T1 significantly changed (*p* < 0.01), so the technique used is immediately effective after resin application for the camouflage of WSLs; furthermore, the results obtained appears to be steady one year after treatment (T1 vs. T2). 

The instrumental colorimetric evaluation between WSL or treated lesions and SAE in every patient agrees with the data present in the literature. Our research methodology allows researchers to describe in a more precise and statistically relevant way the colorimetric variation when comparing the samples and among different groups.

The minimal invasiveness of this technique, attested by other studies and the color variations derived from our investigation, concurs to confirm that the WSL infiltrating technique using TEGDMA in the non-cavitated phase is a valid alternative to traditional restorative techniques.

## Figures and Tables

**Table 1:** Operative procedures.

• Isolation of the treated tooth with a rubber dam
• Removal of the superficial plaque with a nylon prophy toothbrush mounted on a contra-angle
• Etching of the white spot and surrounding healthy enamel with 15% HCL gel for 2′
• Washing with air and water for 30″
• Application of 99% ethanol dehydrating solution for 30″
• The etching process can be repeated for 2′, for a maximum of three if the WSL is still visible
• Drying of the lesion with air
• Application of infiltrating resin for 3′
• Elimination of excess with air
• Light curing for 40″
• Polishing.

**Table 2:** ΔE (WSL vs. SAE) at T0, T1, and T2, and ΔE WSL (T0 vs. T1) for each patient.

Pz.	ΔE WSL vs. SAE			ΔE WSL	Pz.	ΔE WSL vs. SAE			ΔE WSL
	T0	T1	T2	T0 vs. T1		T0	T1	T2	T0 vs. T1
**1**	10,507	4659	7250	5898	**12**	10,550	8060	9117	17,685
**2**	10,546	8060	7864	17,678	**13**	13,302	10,476	11,389	5562
**3**	12,252	4554	3889	11.394	**14**	6425	5578	5644	7.502
**4**	19,054	5678	4994	13,561	**15**	6691	4093	3882	6249
**5**	12,252	4592	3814	11,426	**16**	12,614	3895	4181	11,078
**6**	11,053	10,194	11,083	3923	**17**	10,054	4424	4703	9516
**7**	12,218	5702	5278	8730	**18**	10,442	4637	3488	5886
**8**	6646	4134	3908	6249	**19**	8921	3598	2355	9197
**9**	11,229	5651	4351	9523	**20**	8505	1300	6823	7818
**10**	6590	5165	20,000	4200	**21**	10,321	12,988	13,282	17,395
**11**	19,054	5678	5736	13,561	**22**	9817	3048	1802	6931
